# Contrasting patterns from two invasion fronts suggest a niche shift of an invasive predator of native bees

**DOI:** 10.7717/peerj.13269

**Published:** 2022-05-10

**Authors:** Maria João Verdasca, Luisa Carvalheiro, Jesus Aguirre Gutierrez, José Pedro Granadeiro, Quentin Rome, Sebastien J. Puechmaille, Rui Rebelo, Hugo Rebelo

**Affiliations:** 1 cE3c - Centre for Ecology, Evolution and Environmental Changes, Faculty of Sciences of Lisbon University, Lisboa, Portugal; 2 Departamento de Ecologia, Universidade Federal de Goiás, Goiana, Brasil; 3 School of Geography and the Environment, University of Oxford, Environmental Change Institute, Oxford, UK; 4 Naturalis Biodiversity Center, Biodiversity Dynamics, Leiden, Netherlands; 5 Centre for Environmental and Marine Studies (CESAM) — Departamento de Biologia Animal, Faculdade de Ciências da Universidade de Lisboa, Lisboa, Portugal; 6 UMS 2006 PatriNat –OFB, CNRS, MNHN, Muséum National d’Histoire Naturelle, Paris, France; 7 ISYEB UMR 7205 CNRS MNHN UPMC EPHE, Muséum National d’Histoire Naturelle, Paris, France; 8 Zoological Institute and Museum, University of Greifswald, Greifswald, Germany; 9 School of Biology and Environmental Sciences, University College Dublin, Dublin, Ireland; 10 ISEM, University of Montpellier, Montpellier, France; 11 University of Porto, CIBIO/InBIO, Porto, Portugal; 12 CEABN/InBIO, University of Lisbon, Lisboa, Portugal

**Keywords:** Invasive species, Niche dynamics, Realized niche, Reciprocal distribution models, *Vespa velutina*

## Abstract

**Background:**

The accuracy of predictions of invasive species ranges is dependent on niche similarity between invasive and native populations and on our ability to identify the niche characteristics. With this work we aimed to compare the niche dynamics of two genetically related invasive populations of *Vespa velutina* (an effective predator of honeybees and wild pollinators), in two distinct climatic regions, one in central Europe and another one in the north-western Iberian Peninsula, and hence to identify uninvaded regions susceptible to invasion.

**Methods:**

Niche dynamics and shifts of *V. velutina* were assessed by comparing the environmental niches of the native and of the two invasive populations, using climatic, topographic and land use variables. We also ran reciprocal distribution models using different algorithms and records from both native and invasive ranges to compare model predictions and estimate which regions are at a greater risk of being invaded.

**Results:**

An apparent niche shift was detected in the population of the NW of Iberian Peninsula, where the species is living under environmental conditions different from the native niche. In central Europe, large suitable areas remain unoccupied. The fact that both invasive populations are well established, despite occupying environmentally distinct regions indicates that *V. velutina* has a high ability to successfully invade different environmental envelopes from those existing in its native range. For example, in north-western Iberian Peninsula the species is now thriving out of its native niche limits. Moreover, the large extent of still unoccupied environmental space with similar conditions to those used by the species in its native range suggests that there is still a large area of central and eastern Europe that can be potentially invaded by the species.

## Introduction

The niche concept is broadly used to characterize requirements and impacts of species (Chase & Leibold, 2003), and to predict ecological and evolutionary responses to environmental change (Lavergne et al., 2010). If the niche characteristics of a species in its native range are known to a large extent (they can never be completely inferred from occurrence data studies), it is possible to anticipate with greater precision the potential geographic course of its spread, should the species be introduced in a new area and become an invader (Peterson, 2003). Niches are defined by the species’ physiological tolerances (the fundamental niche which represents the conditions under which a species can live indefinitely), as well as by biotic interactions and dispersal barriers, which constrain the fundamental niche to the realized niche, *i.e.*, the conditions under which a species actually lives (Tingley et al., 2014).

**Figure 1:**
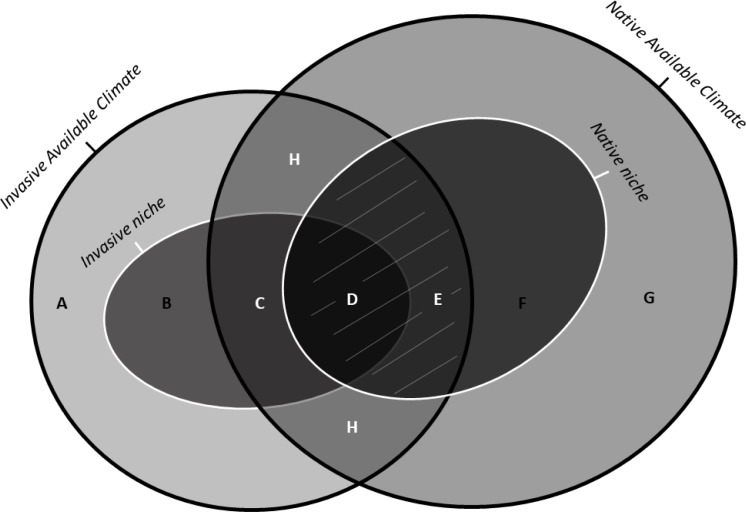
Schematic representation of the indices of niche change (unfilling, stability, and expansion): adapted from Guisan et al. (2014) under the Elsevier License number 5243580352207. Unbroken black lines show the density of available environments in the native range (on the right) and in the invasive range (on the left). The unbroken white lines on the left and on the right show the invasive and the native niches, respectively. The area with white uppercase letters shows the most frequent environments common to both ranges (*i.e.*, analogue environments). The uppercase letters represent: (A) Available conditions in the invaded range but outside of the invasive niche and non-analogue to the native range (B) Novel conditions, *i.e.*, conditions inside of the invasive niche but non-analogue to the native range. (C) Niche expansion, that is, conditions used by the species in the invasive range that, although available in the native range, are not exploited by the native population due to physical barriers or biotic interactions. (D) Niche stability, that is, conditions occuppied in both native and invaded range. (E) Unfilling, that is, conditions inside of the native niche but outside the invaded niche , possibly due to recent introduction combined with ongoing dispersal of the invasive species, which should at term fill these conditions . (F) Conditions inside of the native niche but non-analogue to the invaded range. (G) Available conditions in the native range, outside of the native niche and non-analogue to the invaded range. (H) Analogue conditions containing niche unfilling (E), stability (D) and expansion (C). The grey lines covering the regions (D) and (E) represent the Maximum Niche Stability that a species can occupy in the invaded range if there is no occupation of entirely novel conditions.

Non-native species, when introduced to new geographic areas, may establish in environmental conditions different from their native range because of the lack of natural enemies (*i.e.*, occupying a larger realized niche) or local adaptation (*i.e.*, changing their fundamental niche), or both. Therefore, the analysis of the changes in the niche depicted by an invasive species between its native and introduced ranges may be useful in understanding range expansion and invasion potential (González-Moreno et al., 2014; Kumar et al., 2015). Non-analogue climates represent a severe problem when calculating these niche change metrics, because no insight on the biology of the species in these non-analogue climates can be inferred from a comparison between ranges (Guisan et al., 2014). The colonization of parts of environmental space not present in the native range cannot be unambiguously considered as resulting from evolution (*i.e.*, changes in fundamental niche) in the non-native range. To address this issue, Guisan et al. (2014) proposed a framework for niche comparison between native and invasive ranges with three basic components (Fig. 1): (i) niche unfilling, representing environmental conditions similar to the niche of the native population not (yet) occupied in the invasive range (quantifying niche unfilling areas in the invaded range is critically important because it suggests the likelihood of further spread: González-Moreno et al., 2014); (ii) niche stability, representing overlapping environmental conditions used by the species in both the native and invasive ranges; (iii) niche expansion, representing environmental conditions used by the species in the invasive range that although available in the native range are not exploited by the native population, due to physical barriers or biotic interactions, like competition, predators.

Inspecting marginality is also important to draw inferences about a species invasion ability. Marginality can be defined as the distance between the mean habitat conditions used by the species and the mean environmental conditions over the entire area (Hernández Fariñas et al., 2015). Populations with marginal niches occur in less common environmental conditions in a given area, contrasting with those with non-marginal niches (Hernández Fariñas et al., 2015), which are closer to the centre of the niche hyperspace; see Fig. S1). This means that species with a marginal niche in a given region usually have low tolerance to one or more of the commonest local features, and this can reduce their invasion ability. Hence, by inspecting the marginality of an invasive population we are also adding new information about its potential ability to continue the invasion process.

Ecological niche modelling is often used to anticipate the potential geographic extent of an invasive process. In particular, ensemble forecast predictions (Araújo & New, 2007) have been frequently used in invasive ecology, as they represent a consensus approach that offers more robust predictions for the potential and realized distribution of species than techniques that rely on a single algorithm. Reciprocal distribution modelling (RDM) emerged as a powerful tool by considering both the invasive and native ranges in distribution models (Medley, 2010); otherwise, the potential range of a species may be seriously underestimated (Václavík & Meentemeyer, 2012). Niche conservation is indicated if both the native model accurately predicts non-native distributions, and non-native models accurately predict the native distribution (Medley, 2010). Alternatively, a niche shift is suspected whenever reciprocal models poorly predict one another, although that situation can also be caused by a narrower niche in the invasive range. Indeed, this RDM approach has previously revealed niche shifts for the spotted knapweed and fire ant invasions in the United States (Broennimann et al., 2007; Fitzpatrick et al., 2007). In a recent work by Hill, Gallardo & Terblanche (2017), the authors suggest that distribution models should not be used in isolation to predict insect invasions or invaded range extents, but instead need to be coupled at least with some analysis of changes in environmental/climatic space.

**Figure 2:**
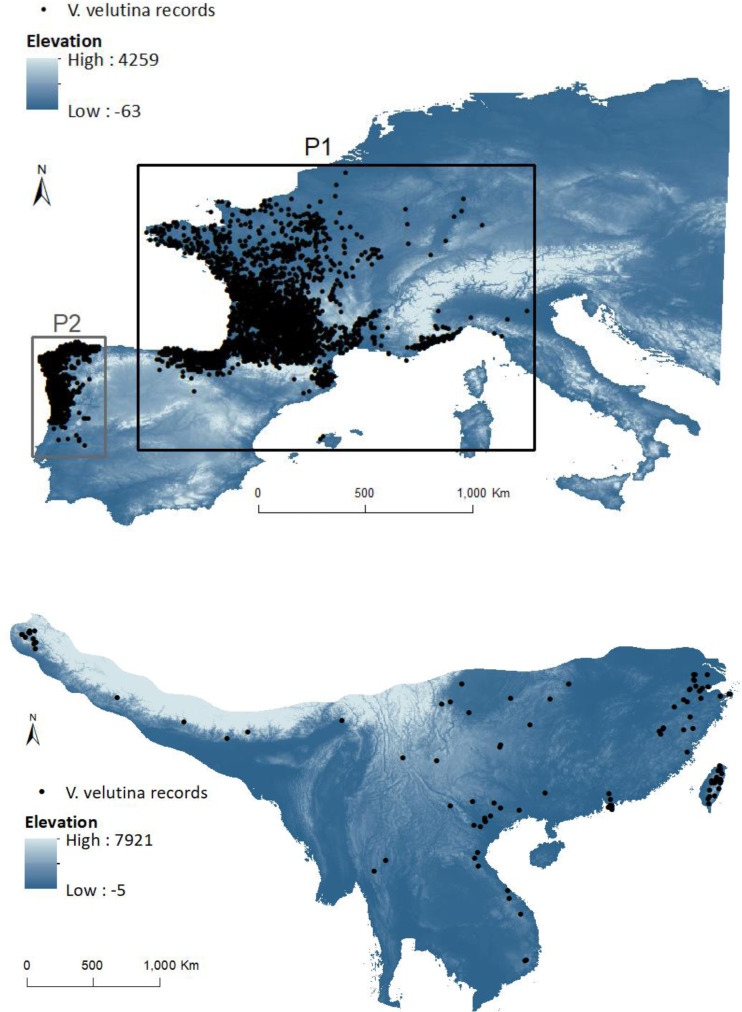
Invasive (above) and native (below) ranges of *Vespa velutina*. European study area (with elevation in background) includes the two invasive subpopulations: P1 (black box containing the population from France and contiguous regions of Italy and Spain) and P2 (grey box containing the population from NW of Iberian Peninsula). The native envelope was delimited by the area that encompasses the different closely related subspecies in the native range.

Many invasive insects are not in equilibrium in their invasive ranges, and have the potential to further expansion (Hill, Gallardo & Terblanche, 2017). As invasive insects can respond quickly to novel environments (including biotic interactions) through either phenotypic plasticity, changes in adaptive traits or some combination of both (Urbanski et al., 2012; Gibert et al., 2016), niche shifts may be relatively common within this group. Frequently a single species invades several regions, and this provides an opportunity to study and model its invasion ability, particularly to assess potential niche shifts. One of the most recent examples of a successful invasion in Europe is the yellow-legged hornet, *Vespa velutina* Lepeletier, 1836. Native to south-east Asia, *V. velutina* is a predator of honeybees and other beneficial insects (Monceau, Bonnard & Thiéry, 2014; Rome et al., 2021; Verdasca et al., 2021a), being considered a serious threat to honey production, pollination services and consequently food security (Monceau, Bonnard & Thiéry, 2014; Requier et al., 2019; Verdasca et al., 2021b). The mere presence of *V. velutina* near beehives induces stress to honeybees (Leza et al., 2019) and decreases their foraging activity, thus reducing pollen and nectar collection, with impacts on winter survival of the colony (Requier et al., 2019). Further, as *V. velutina* can attack a broad variety of wild pollinators, it could also pose additional negative effects on crop production as well as on biodiversity (Monceau, Bonnard & Thiéry, 2014). Since the accidental introduction in France, in 2004 (in a temperate bioclimatic region: Sayre et al., 2020) of a single female fertilized by several males (Arca et al., 2015), *V. velutina* has spread to other European countries, being considered an invasive alien species of concern in the European Union (European Commission, 2016). In 2011, a new invasion by *V. velutina* was found in the North of Portugal (Fig. 2–P2) in the region of Viana do Castelo (Grosso-Silva & Maia, 2012), near a paper mill, where the species probably arrived in a load of timber from France (Marco Portocarrero, pers. comm., 2018), in a transitional region between a temperate hyper oceanic climate to the north and a Mediterranean climate to the south; the latter conditions with no apparent parallel with any bioclimatic region of the native range (Sayre et al., 2020). The new invasive population has been expanding south into central Portugal (Verdasca et al., 2021c), and to the north and east into Galicia (Spain), where it arrived at the end of 2012 (Rodríguez-Flores et al., 2019).

Genetic analyses of individuals from different countries of the invaded area (France, Spain, Portugal, Italy, and UK) confirm a low genetic variability of *V. velutina* in Europe and that they all derived from the spreading (natural or human mediated) of the invasive population initially established in France (Fig. 2–P1) (Budge et al., 2017; Granato et al., 2019; Quaresma et al., 2022). Founding of P2 from P1 was accompanied by a reduction in the species genetic diversity (Quaresma et al., 2022), but this did not hamper the successful spread of *V. velutina* in Portugal (Carvalho et al., 2020; Verdasca et al., 2021c).

Recently, Barbet-Massin et al. (2018) suggested that the climatic niche of *V. velutina* in France expanded during the past few years, raising the hypothesis of a change in the niche dynamics during the invasion process. However, their model was not able to predict some of the already invaded areas in Portugal and Germany. Here, we expand on this previous work to assess niche shifts in a newly invaded region, the NW of Iberian Peninsula, and compare them to the realized niche seen in the older invasive population in France, incorporating the impacts of time since invasion on niche metrics. Specifically, we compared the realized niche of these two genetically related populations of *V. velutina* in Europe and coupled niche dynamic analysis and RDM with an ensemble forecast to address three main questions: (i) Has *V. velutina* shifted its niche during the invasion process? (ii) Is *V. velutina* occupying the most common environmental conditions of the invasive range? (iii) Is there potential for further expansion? Answering these questions is crucial to understand niche flexibility and its potential for evolution in different regions of the invaded range, to identify areas at risk, and to inform management plans.

## Material and Methods

### *Vespa velutina* occurrence data

Distribution data sets consisted of presence data only. We enriched the native dataset used by Barbet-Massin et al. (2018) (that only used the subspecies *Vespa velutina nigrithorax*), by considering the different subspecies (see Perrard et al., 2014) present in Asiatic mainland (148 records). With the current fragmented knowledge in Asia, none of the different subspecies is geographically well covered in the native range being very difficult to discriminate the spatial limits of each population. A recent work by Takeuchi et al. (2017) showed a clear divergence between the Malaysian and Indonesian populations and all the other Asian mainland (and Taiwanese) populations and colour morphs, while also showing that the haplotypes from these continental and Taiwanese forms do not clearly segregate geographically. Genetic exchange is therefore probably still occurring across all the native mainland populations and the validity of several subspecies and colour morphs is questioned; therefore, the records from the native range used here refer to all the records, regardless of the putative subspecies, except for the Indonesian and Malaysian populations (Takeuchi et al., 2017). Although there are considerably less records from the native range available (in comparison with invasive range: see below), they capture the entire environmental envelope where the species thrives, being so far the most complete dataset used to model this species.

For the invasive range we gathered a total of 20,855 records from different sources (for details see Supplemental Information). The effort was not systematic once it results from citizen reports, although all the records were previously validated by the different entities that hold the data. Since by 2017 the records were not continuously distributed in Europe it was possible to identify two different populations that had not yet come into contact in the North of Spain (data provided to us in 2017 by the Spanish Ministerio de Agricultura y Pesca, Alimentación y Medio Ambiente). Hence, it was possible to attribute each location to either the first introduction in France or the second introduction in Portugal. To check for commonalities in the set of factors affecting the distribution of *V. velutina* within Europe, we divided the records in two sets: P1 with data from France and contiguous records from Italy and NE Spain; P2 in the NW of Iberian Peninsula, with records from Portugal and from the contiguous Galicia (Fig. 2). We could ascribe a date for 81.9% of the European data. To analyse the variation of realized niche metrics with time since introduction (see methods below), these data were grouped by year (the remaining 18.1% of the data were discarded only for this specific analysis). We compared each year with the combination of all the previous years.

To avoid spatial autocorrelation, we reduced the number of occurrence data through the “spatially rarefy occurrence data” tool in SDMtoolbox (Brown, 2014) weighed by a principal components analysis (PCA) on climatic data, thus keeping the points with unique environmental information (in a 5 km pixel resolution); this resulted in a total of 110 points for Asia and 2,582 for Europe and (Fig. 2; Appendix S1).

### Environmental data

We considered an initial dataset of 22 environmental variables (climatic, topographic and land use: ‘see Table S1 for details’) which potentially affect the ecophysiology of *Vespa velutina* (Villemant et al., 2011; Bessa et al., 2016). We calculated the distance to each land cover type since distance variables are acknowledged to achieve a better performance for species associations to landscape features (Rainho & Palmeirim, 2011). As nests are made in trees, but also in the ground, on walls, on slopes with vegetation, we decided to test for a number of topographic components (slope included). All the layers were clipped to the same extent and upscaled to a 5 km pixel resolution, to match known *V. velutina* homing ability (Poidatz et al., 2018). Climatic data of the available environmental space and of the occupied niche in both native and invasive ranges were compiled to compare its range of variation (’see S4’). All calculations regarding the preparation of variables for analysis were carried out in ArcGIS 10.4.1 software (ESRI 2016). To avoid high collinearity, variables that were highly correlated (r ≥ 0.70: Dormann et al., 2013), examined on both ranges, see Tables S2 and S3) were excluded from subsequent analysis, keeping the ones with higher acknowledged biological importance. With the remaining twelve variables (Table 1), a multivariate environmental similarity surface (MESS) calculated in MaxEnt, spatially identified which areas in the invasive range possess similar environmental characteristics to the native range (see Fig. S2). The native range was delimited to contain only the regions where the species is acknowledged to live (we took care to not include areas with extreme climates that have no parallel in Europe, like the cold regions of Himalayas or the tropical regions of Malaysia). *V. velutina* is still expanding in the invasive range, thus to estimate its potential distribution we used three different non-parametric presence-only algorithms (please see below) using the ‘biomod2’ package (Thuiller, Georges & Engler, 2014) in R (R Core Team, 2019).

**Table 1:** Variables used in the modelling of the realized niche of *Vespa velutina* (for details of resolution and source of each variable, see Table S1).

**Type**	**Variables**
**Climatic**	BIO1 = Annual Mean Temperature
	BIO7 = Temperature Annual Range (BIO5-BIO6)
	BIO10 = Mean Temperature of Warmest Quarter
	BIO11 = Mean Temperature of Coldest Quarter
	BIO17 = Precipitation of Driest Quarter
	BIO19 = Precipitation of Coldest Quarter
**Topography**	slp = Slope
	northn = Northeness
	eastn = Eastness
**Land cover**	durb = Distance to urban areas
	dfor = Distance to forest
	dwat = Distance to water

**Figure 3:**
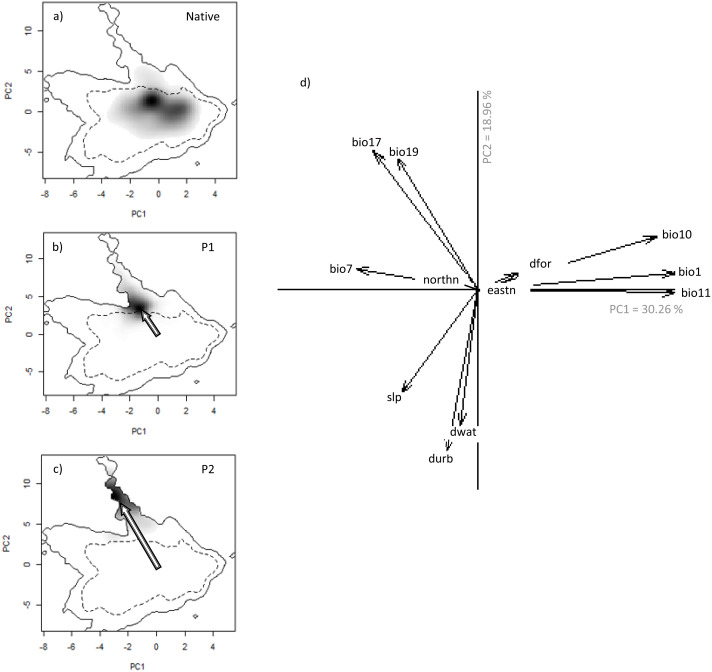
Niche of *V. velutina.* in environmental space of a principal component analysis (PCA-env). The left three panels represent the niche of the species along the two first axes of the PCA in: (A) the Asiatic native range; (B) in the invaded range of subpopulation P1 (from France and contiguous regions of Italy and Spain) and (C) in the invaded range of subpopulation P2 (from NW of Iberian Peninsula). Grey shading shows the density of the occurrences of the species by cell. The solid and dashed contour lines illustrate, respectively, the kernel density estimates corresponding to 100% and 50% of the available (background) environment. The arrows represent how the centre of the niche has changed between Asian native and European invaded range. (D) Contribution of the environmental variables on the two axes of the PCA (Climatic: bio1—Annual Mean Temperature; bio7—Temperature Annual Range; bio10—Mean Temperature of Warmest Quarter; bio11—Mean Temperature of Coldest Quarter; bio17—Precipitation of Driest Quarter; bio19—Precipitation of Coldest Quarter; Land cover: dfor—Distance to forest; dwat—Distance to water; durb—Distance to urban; Topography: eastn—Eastness; northn—Northness; slp—slope) on the two axes of the PCA and the percentage of variance explained by the two axes.

### Has *V. velutina* shifted its niche during the invasion process?

To test this hypothesis, we used the R package ‘ecospat’, which can be applied to investigate the niche of invasive species, by using an ordination method to quantify and compare niche shifts between invasive and native ranges (Di Cola et al., 2017). We performed two complementary analyses:

(i) We compared the environmental niche space between native and invasive ranges of *V. velutina*. We performed a PCA to summarize the selected environmental variables; this was calculated using environmental values from all the pixels of both the native and the invaded areas, thus maximizing the ecological variance. We then applied a kernel density function of the occurrence data over the PCA space, to estimate the density of the occurrences of the species by cell (Broennimann et al., 2012; Di Cola et al., 2017; Fig. 3). We plotted all datasets (native, P1 and P2) on the environmental space and visually analysed them for niche shifts between native and invasive populations. The niche equivalency (*i.e.*, whether the niche overlap is constant when randomly reallocating the occurrences of both entities among the two ranges) and niche similarity (addressing whether the environmental niche occupied in one range is more similar to the one occupied in the other range than would be expected by chance) were tested in ‘ecospat’ (see Broennimann et al. (2012) for methodological details).

(ii) We inspected temporal niche dynamics for each focus of invasion (2006–2015 for P1 and 2014–2017 for P2). Based on the three niche metrics (Expansion, Unfilling and Stability) defined by Guisan et al. (2014), we decided to rename the metric “Stability” into “Maximum Niche Stability”, to facilitate the interpretation of the results, since the Unfilling metric also corresponds to stability conditions that are not (yet) filled. Niche overlap was then decomposed across time (yearly) in the three metrics of niche (corresponding to the areas E, (D+E) and C; Fig. 1). This decomposition provides more information about the drivers of niche dynamics between native and invaded ranges (Petitpierre et al., 2012; Guisan et al., 2014). These calculations only took into account areas with analogous environmental conditions (Qiao, Escobar & Peterson, 2017) enabling the correction for differences in the availability of environments between study areas and preventing the detection of false niche shifts. We thus avoided the inclusion of environmental conditions that occur in the native but not in the invasive ranges (Petitpierre et al., 2012), restricting niche analyses to the intersection between native and invaded ranges in the PCA (areas represented by (C), (D) (E) and (H) on Fig. 1). Schoener’s D index (ranging between 0 and 1) was used as a measure of niche overlap (Petitpierre et al., 2012). By comparing niche overlap between a given year and all the years prior to that accumulated as one, we then evaluated the dynamics of the realized niche in the invasive range.

### Is *Vespa velutina* occupying the most common environmental conditions of the invasive range?

For each invasive population we performed a theoretical exercise in the R package ‘ecospat’ (Di Cola et al., 2017) to inspect for changes in niche metrics when marginal conditions were discarded from the analyses, enabling this way to infer about the use of rare environmental conditions (for details how the niche metrics works please see: Guisan et al. (2014)).

We repeated the calculations of the niche metrics, discarding successively an increasing proportion (10% and 20%) of the most marginal (*i.e.*, less frequent) available environmental conditions. We progressively reduced the “intersect” parameter (quantile of the environmental density used to remove marginal climates), removing up to 20% of the area with analogous conditions between both invasive and native ranges (area in Fig. 1H). When the “intersect” parameter was set to zero the analysis included all analogue environmental conditions between native and invaded range. By changing the same parameter to 0.1, we excluded 10% of the marginal climate, being the analysis restricted to 90% of the overlap between native and invaded environmental space, and so on, until 20% of the most marginal environmental conditions had been removed. With this, we aimed to infer if the two invasive populations of *V. velutina* are occupying the most frequent or rather distinct (*i.e.*, marginal) environmental conditions occurring at both invasive and native ranges. If a low variation in the niche metrics is detected after excluding marginal climates from the analysis, it means that the species is not exploring conditions that are rare in the study area. Conversely, if the exclusion of marginal climates leads to a large variation in the niche metrics, it means that the species is using those uncommon conditions, hence its populations are less prone to further expand.

### Is there potential for further expansion?

We used a RDM approach (Medley, 2010; Broennimann et al., 2012) to evaluate *V. velutina* expansion potential to uninvaded European areas and to identify which regions may be at higher risk of invasion. For this RDM approach, we fitted the species distribution models (SDMs) using data (i) from the native range to fit the first set of SDMs models, (ii) from France and contiguous regions of Italy and Spain invaded range (P1) and (iii) from NW of Iberian Peninsula (P2) invaded ranges. We applied three commonly used non-parametric algorithms: Generalized Boosted Models (GBM; Friedman, 2001), Random Forests (RF; Breiman, 2001) and Maximum Entropy modelling (Maxent; Phillips, Anderson & Schapire, 2006) (for details see Supplemental Information). For each one of the datasets (full, P1 and P2) we fitted 10 model repetitions per algorithm using 75% of the data for modelling training and 25% for model testing, creating the number of pseudo-absences (background data for MaxEnt) as five times the number of presences used. We thus produced 30 spatially explicit predictions (10 model repetitions ×3 algorithms) per dataset. We converted the continuous model suitability scores to binary predictions using the thresholds that maximize the sensitivity and specificity of the models (Liu, White & Newell, 2013) and then created a final ensemble model per dataset (full, P1 and P2). For the ensemble models we considered a presence only when more than half of the models predicted a cell as presence (Araújo & New, 2007). We used the area under the ROC (Receiving Operating Characteristic) curve (AUC) (Phillips, Anderson & Schapire, 2006) to evaluate model performance. The AUC is not only threshold independent but also evaluates both the false-positive error rate and the true positive rate in order to obtain a measure for the accuracy of the constructed model (Aguirre-Gutiérrez et al., 2013). We obtained AUC values from each of the models created by the 10 repetitions for each algorithm. Currently the AUC is one of the most used methods for model evaluation (Razgour et al., 2016).

To identify the regions in Europe with environmental conditions similar to the native range, we overlapped the models (from native to invasive and from invasive to invasive) previously produced, highlighting the areas of agreement between environmental conditions of both ranges. High-risk areas were identified as the regions predicted by both native and invasive models, or only by one of the models. Low risk areas were predicted to be unsuitable by both models.

## Results

### Has *Vespa velutina* shifted its niche during the invasion process?

We detected evidence of a niche shift (within the realized niche of *V. velutina*) in Europe, especially in the invasive population from the NW of Iberian Peninsula (P2) towards climatic conditions different from those of the native range (3% overlap with the native niche—Fig. 3C). For the French population (P1), the species realized niche is partially nested in environmental conditions similar to the ones used in the native range (overlap of 30% with the native range). In the NW of Iberian Peninsula, the species thrives under different environmental conditions from those used by the species in France (Figs. 3B, 3C). Among the environmental variables considered, climatic variables related with precipitation were the ones that better explained the probability of invasion by *V. velutina* (Fig. 3D). Niche equivalency was rejected for both populations, indicating that both suffered alteration of their realized niche during the invasion process. However, and for both populations, niche overlap falls within the 95% confidence limits of the null distributions, meaning that niche similarity cannot be rejected. (see Figs. S3 and S4).

Analysing the niche dynamics across years following invasion, our results suggest that at the beginning of the invasive process, P1 population was using both expansion and stability conditions, being 97% of the environmental conditions similar to the native niche not yet occupied (Table 2 –Unfilling; corresponding to region Fig. 1E). This proportion has steadily diminished to ∼65% , after 9 years (Table 2). In the NW of Iberian Peninsula (P2), from the very beginning the species expanded into environmental conditions that are apparently not explored in the native range (Table 2, corresponding to region in Fig. 1C). This proportion has been rather stable over the years.

**Table 2:** Niche dynamics in Europe. Variation of niche metrics (Expansion, Maximum Niche Stability and Unfilling) along the years in the two sets of invasive subpopulations (P1—from France and contiguous region of Italy and Spain; and P2—from NW of Iberian Peninsula) and corresponding overlap between native and invaded ranges. The uppercase letters (C), (D) and (E) correspond to the regions presented in Fig. 1.

**Population**	**Year**	**Expansion (C)**	**Maximum Niche Stability (D) + (E)**	**Unfilling (E)**	**Overlap (Schoener’s D index)**
P1	2006	0.63	0.37	0.97	0.18
	2007	0.53	0.47	0.91	0.20
	2008	0.55	0.45	0.89	0.18
	2009	0.51	0.49	0.82	0.19
	2010	0.47	0.53	0.79	0.21
	2011	0.43	0.57	0.72	0.24
	2012	0.41	0.59	0.70	0.25
	2013	0.39	0.61	0.70	0.27
	2014	0.36	0.64	0.67	0.29
	2015	0.34	0.66	0.65	0.30
P2	2014	1.00	0.00	1.00	0.00
	2015	0.98	0.02	0.99	0.01
	2016	0.99	0.01	0.99	0.00
	2017	0.99	0.01	0.99	0.00

### Is *Vespa velutina* occupying the most common environmental conditions of the invasive ranges?

Reducing in 20% of the environmental space with analogous conditions between both invasive and native ranges (to exclude marginal climates from the analyses) did not lead to a large variation in the niche metrics in both populations (Table 3). This result suggests that the species is not occupying marginal (*i.e.*, uncommon) environmental conditions in Europe. Instead, the species is occupying the most common conditions available.

**Table 3:** Effect of marginal environmental conditions on the three niche indices: expansion, maximum niche stability and unfilling. The metrics were calculated by reducing the intersection parameter from 0% to 20%. Global overlap between native and invaded ranges is also shown.

**Population**	**Intersect**	**Expansion (C)**	**Maximum Niche Stability (D)+(E)**	**Unfilling (E)**	**Overlap (Schoener’s D index)**
P1	0	0.34	0.66	0.50	0.29
	0.1	0.29	0.71	0.50	
	0.2	0.20	0.80	0.50	
P2	0	0.92	0.08	0.98	0.03
	0.1	0.86	0.14	0.98	
	0.2	0.74	0.26	0.99	

### Is there potential for further expansion?

Before modelling procedures, we checked for the existence of analogous conditions between native and invasive ranges. The MESS analysis showed that almost 100% (>99.99%) of the invasive range did not have one or more environmental variables outside the range present in the training data (Fig. S2), enabling the predictions for these areas. Regarding the predictive models for the invasive range, AUC values were high (all over 0.93) for the different algorithms, although for the native model the AUC values ranged between 0.73 and 0.91 (Fig. S5). The model with highest fit was obtained using RF (higher AUC values than GBM and Maxent). Comparing model sensitivity to small changes in occurrence points and using the model developed for the invasive range, the predicted area for the species in Europe was relatively similar for the different algorithms but with larger within-model variance for Maxent and RF (see Fig. S6A). For the projection of native occurrences into Europe, the sensitivity to small changes in the presence data varied more between algorithms, being Maxent the more susceptible regarding model variance (see Fig. S6B).

**Figure 4:**
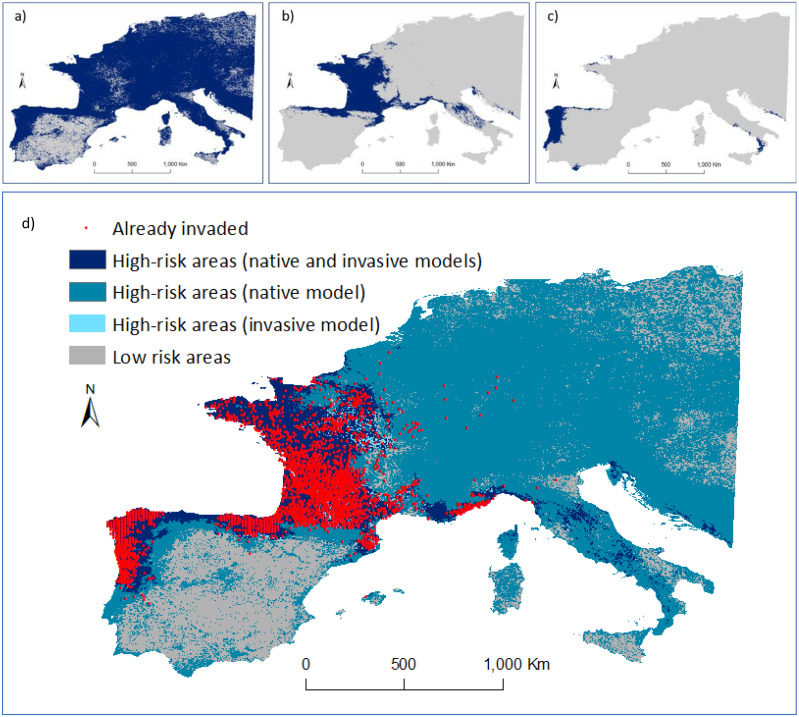
Predicted distribution of *Vespa velutina* in Europe. (A) Ensemble model using data collected in the native range (prediction area = 3,467,700 km^2^); (B) ensemble model using data from P1 alone (from France and contiguous regions of Italy and Spain; prediction area = 793,675 km^2^; (C) ensemble model based on P2 data alone (from NW of Iberian Peninsula; prediction area = 156,675 km^2^). Distribution models were calculated in ‘biomod2’, and then converted into binary predictions using their correspondent thresholds that maximize the sensitivity and specificity of the models. Every cell for which more than half of the models predicted a presence, was considered a presence, the other cells were assigned as absences. (D) Risk map highlighting the areas of agreement between the models of the native and invasive ranges of *V. velutina*.

When only the P1 or P2 invasive ranges were considered for the modelling procedure, the full extent of areas already invaded were not detected (Figs. 4B, 4C). However, the native range model overlapped and covered most occurrences in the invasive range (Figs. 4A and 4D). As the native model identified 99% of the high-risk areas, we considered these as the regions that are not (yet) occupied despite having similar environmental conditions to those used by the species in the native range. In contrast, the predicted presence of the species in Asia using the model developed for Europe did not accurately predict its native distribution.

An agreement between native and invasive models occurred for only 15% of the potential distribution in the invaded range. The remaining areas of concordance between both models that are still unoccupied by the hornet are in the south of Italy, Corsica, Croatia and Montenegro. About 65% of the area where the species is predicted to occur in Europe was considered at high-risk of being invaded, as it represents similar environmental conditions to those found on *V. velutina’*s native range, though this area remains uninvaded (Fig. 4D). Concerning the presence records, all were located on high-risk areas. At this continental scale, the climatic variables related with precipitation and temperature contributed the most to explain the distribution of *V. velutina* (Fig. S7). More specifically, the species is thriving in Europe in regions where the winters are mild and rainy, and the summers are neither too hot nor too dry.

## Discussion

Our results suggest that a shift in the niche of *V. velutina* occurred in Europe, probably in the NW of Iberian Peninsula, and that it is essential to consider both native and invasive records in modelling procedures to accurately predict the species invasive potential. Our study also revealed that the colonization process by *V. velutina* is still ongoing with a high potential for geographical expansion, as a large extent of Europe with analogue environmental conditions to those of the native niche is still unoccupied. The fact that the area we predicted to be suitable for *V. velutina* is larger than in previous studies, makes this the first study to accurately predict all the currently invaded area in Europe (even the very recent records in Hamburg, Germany, and the southwestern occurrences in the Iberian Peninsula): this was due to the innovative approach here conducted - coupling reciprocal distribution models with niche analysis; and using of all *V. velutina* occurrences in the native range, irrespective of the different colormorphs (except for the clear divergent forms of Indonesia and Malaysia), allied with the use of the largest dataset so far to model this invasive. Further, we also evidenced that this species is using the most frequently available conditions in Europe, hence is less prone to possible stochastic events that could hamper its progress.

Describing species’ niches and understand whether these can change rapidly (niche shifts) or not (niche conservatism) between different geographic areas or time periods is important to anticipate invasions in the context of ongoing global change (Guisan et al., 2014). A genuine shift in the fundamental niche would reflect evolutionary adaptation of a species to novel environmental conditions, whereas occupying new portions of the same fundamental niche does not change the environmental potential of the species (Qiao, Escobar & Peterson, 2017). Regardless of the clearly different environmental conditions occupied by the two European invasive populations chosen for this work, *V. velutina* is well established in both regions.

Despite some variability across taxa, a recent review with over 400 species showed limited niche expansion between native and introduced ranges, supporting the niche conservatism hypothesis (Liu et al., 2020). However, in our study, niche expansion contributes respectively to 34% (P1) and 92% (P2) of the global population niche, meaning that this species can successfully occupy different environmental conditions from those occupied in the native range. Differences between environmental conditions in the native range and the P2 invaded range (suggesting niche shift) were particularly clear (only 3% of niche overlap). However, as the analyses of niche dynamics are necessarily restricted to the environmental space with analogue conditions between native and invaded ranges (region Fig. 1H), we cannot fully assess the use of novel conditions (*i.e.*, that are not available in the native range) in the invaded range. Still, we can state that, within the same available conditions, our estimate of the realized niche of the species in the Iberian Peninsula is different from its equivalent realized niche in its native range, and that the ability to occupy obviously different realized niches may help explain the species invasiveness.

As recently introduced species do not have enough time to spread to regions far from the introduction site, their occupied climatic space in the introduced range would always be expected to be much smaller than older introductions, and the unoccupied climatic space would gradually decrease in the process of colonization (Petitpierre et al., 2012; Strubbe et al., 2013). Indeed, in France, the invasion began under environmental conditions of expansion and stability, and then the species has been progressively colonizing the unfilled environmental space. Interestingly, in a very short time (less than ten years) it is possible to measure significant changes in niche unfilling metric of this P1 population. However, it is also possible that other invasive populations (like P2) use totally different environmental conditions from those of the native niche right from the very beginning of the expansion process. This supports the high invasive potential of the species. For both populations, *V. velutina* experienced measurable changes in environmental niche occupancy but as would be expected, both invasive niches tend to be more similar to the native niche than would be expected by chance, and niche similarity could not be rejected.

Similar to previous studies (Villemant et al., 2011; Bessa et al., 2016; Fournier et al., 2017; Barbet-Massin et al., 2018), we identified climatic variables as the ones that most contributed to the current distribution of *V. velutina*. Regardless of the adequacy of particular environmental conditions for a species, if they are uncommon on a given area the species can still be limited in its potential to further expand. In our case, by addressing species marginality, we showed that *V. velutina* is using the most common environmental conditions available in Europe. This suggests that the species has all the necessary conditions to continue its expansion process. The increasing high values of the metric Maximum Niche Stability for P1, even when marginal conditions were removed from the analyses, means that the occupied conditions of this population overlap with the core of the species’ realized niche in its native range. In contrast, from the very beginning, the population of the Iberian Peninsula thrives on conditions that are not exploited in the native range.

Niche theory tackles a variety of issues, including evolutionary processes, competition and predation dynamics (Hirzel & Lay, 2008). Hence, the apparent niche shift of the P2 population must be interpreted with caution. Although the European environmental envelope of the species greatly overlaps with the native one, there are some conditions of the realized niche in the invasive range that are not explored in the native. Therefore, the shift may have resulted from several factors: alleviation of biotic constraints and geographic barriers, adaptation to novel conditions, or under sampling of the species in its native range. Invasive species frequently experience release from biotic interactions and dispersal barriers in their invasion process (Colautti et al., 2004). Biotic constraints in the native range that are not present in the invasive one (*e.g.*, presence of efficient predators, parasitoids and pathogens, or competition with other hornets for food resources or nesting sites) will contribute to the expansion of *V. velutina* to conditions not explored in the native range. Indeed, while the invasive *V. velutina* might compete with *Vespa crabro*, for food resources (Cini et al., 2018), reproductive females of *V. velutina*, have a higher pathogen resistance than their native counterpart, which might represent a key factor for the ecological success and spread of this invader (Cappa et al., 2021). Further, if the native dataset is not representative of the full Asian distribution, the entire native environmental envelope might not have been fully captured. This indication of a niche shift may also be simply stochastic (once it depends on the available environmental conditions of the invaded range), or due to a partial filling of the potential native niche in the invaded range, not representing a “true” niche change (Strubbe et al., 2013). The most likely scenario is a realized niche shift in P2 population; however, without experimental evidence it is impossible to elucidate the mechanisms driving the observed shift. It is also possible that the species is rapidly evolving and adapting to the novel conditions in the Iberian Peninsula. Indeed, a recent review by Sherpa & Després (2021) of insect invasion studies showed several examples of post-introduction adaptation and niche shift in invasive insect populations, despite their recent single-source introduction events (see also the work by Sherpa and collaborators (2019) focused on the Asian tiger mosquito *Aedes albopictus*). Although a low level of genetic diversity was detected in the European populations of *V. velutina* (Arca et al., 2015; Granato et al., 2019; Quaresma et al., 2022), this is not necessarily related with inbreeding depression and, in some cases, may lead to local adaptations (Valladares et al., 2014). Further studies on the morphology, ecophysiology, genetic variability, admixture, adaptation, and plasticity of the European populations will elucidate on their degree of local differentiation.

We detected suitable conditions for *V. velutina* in Europe in areas much larger than those estimated by prior prediction exercises (Ibáñez Justicia & Loomans, 2011; Villemant et al., 2011; Fournier et al., 2017; Barbet-Massin et al., 2018; Barbet-Massin, Salles & Courchamp, 2020; Kim et al., 2021). This is particularly relevant for central and eastern Europe where our models have identified, for the first time, suitable conditions for *V. velutina*. This was expected, as this species is still expanding. As we used a higher amount of available environmental information compared to previous studies, our predictions of expansion potential of *V. velutina* are expected to better reflect the environmental space used by the species (Raes, 2012; Carretero & Sillero, 2016). We are aware that the fact we have used the realized native niche (we do not have information on the full fundamental native niche) does not guarantee that all the suitable areas for the species were identified. In fact, most of the already invaded area in Portugal and the new record of *V. velutina* in Hamburg (Husemann et al., 2020) occurred in regions that recent studies identified as unsuitable for the hornet (Fournier et al., 2017; Barbet-Massin et al., 2018; Barbet-Massin, Salles & Courchamp, 2020). The fact that our model has predicted for the first time all the current invaded area in Europe, supports the validity of the risk map provided in this work. Barbet-Massin and collaborators (2018) argue that, to predict the hornet short-term potential of expansion, it is better to run species distribution models without accounting for native data. However, such an approach may not capture the entire environmental envelope where the species is able to thrive. In fact, by using only native occurrences, we predicted a large unoccupied extent with suitable conditions for the species in Europe. Another explanation for the narrower expansion area predicted by Barbet-Massin et al. (2018) is that their study is mostly based on the data from France (corresponding to P1) which used environmental conditions that are relatively similar to those found on the core of the species’ native niche. Our study clearly demonstrates that using only part of invasive records has limited predictive power. On the other hand, as the hornet is still expanding and the realized niche is not stable, the invasive model failed to predict most of native occurrences. This may also be an indication that the niche in Europe is currently changing.

The genetic paradox of invasions suggests that the success of invasive species may result from rapid neutral and even adaptative evolutionary changes that do not require high genetic variability (reviewed by Schrieber & Lachmuth, 2016. During an invasion the gene pool of the new population is likely much smaller than in the source population (Arca et al., 2015). However, Garnas et al. (2016) showed that founder effects rarely limit fitness in invasive insects and may in fact be beneficial by purging harmful alleles or increasing additive genetic variance. Indeed, the European invasive process originated from a single founder female in France, and was followed by a second colonization process in the north-western Iberian Peninsula, where a second founder effect occurred (Quaresma et al., 2022). The establishment of P1 and P2 populations and their ongoing expansion, evidence that a related genetic constitution enables the successful invasion of two climatically distinct regions. The different environmental conditions in the Atlantic coastal zone of Iberian Peninsula did not seem to have hampered the invasion process (Verdasca et al., 2021c). Given that suitable climatic conditions exist and due to the high dispersal ability of this species, the primary front has being expanding throughout the north Atlantic coast in Spain all the way to Galicia, where it recently met the secondary front from Portugal (Quaresma et al., 2022). A substantial gain in the genetic diversity of the Iberian populations ensued, most probably through the incorporation of first-generation migrants both from P1 and P2 populations (Quaresma et al., 2022). This gene-flow can alleviate the negative effects of bottlenecks and bring new allelic combinations and may be contributing to increase population fitness and boost the invasion ability of the species. Therefore, potential genetic adaptation underlying niche shift cannot be completely excluded in the Iberian populations of *V. velutina*.

Apparently, *V. velutina* has a high ability to cope with the challenges presented by the novel European abiotic and biotic features, but the lack of competitors or the enemy release hypothesis (Keane & Crawley, 2002) can also justify its success. Little is known about the composition of natural enemies’ communities (*e.g.*, predators, parasites) and their potential importance on the regulation of *V. velutina* populations. However, in the Asian tropics and subtropics this hornet faces competition with six other hornet species while in Europe it only competes with *Vespa crabro* (Choi, Martin & Lee, 2012). Despite the great overlap in trophic preferences between *V. velutina* and *V. crabro* (Cini et al., 2018), a recent study in a mountainous region in Italy showed that native Vespidae are probably avoiding or minimizing the competition pressure due to their ability to take refuge at high altitudes (>600 m) or to their later life cycle when compared to *V. velutina* (Carisio et al., 2020). Identifying such biotic constrains in the native region could be important to predict (and potentially avoid) new invasions, providing essential information for the development of control strategies.

To develop cost-effective strategies on managing invasions, more attention should be paid to surveying and controlling the spread of invasive species into climatic conditions that are occupied in their native range but (not yet) colonized in their introduced ranges (Guisan et al., 2014). Our results suggest that the invasion process of both invasive populations is likely to continue towards other European countries and that the centre, east and centre-north of Europe are at a high risk of invasion. Otherwise, our models jointly with the expansion sequence so far, and with the temperature and precipitation characteristics of the current colonized areas seem to confirm that *V. velutina* distribution is constrained by climatic factors, namely a need for high precipitation and a relatively narrow range of annual temperatures (Balmori, 2015; Verdasca et al., 2021c). Hence, the expansion of this invasive may be hampered in areas of the Iberian Peninsula subject to a marked thermo-Mediterranean climate (Bessa et al., 2016; Verdasca et al., 2021c). According to Villemant et al. (2011), other suitable regions for *V. velutina*, located in north and south America and in Australia, may also be vulnerable to invasion if another accidental introduction occurs in these continents. The fact that few (9%) records of the invasive population are in high-risk areas (that were only predicted by one of the models—native or invasive) is not surprising, since the invasion started recently. In 2017 very few occurrences were detected in British Isles (not dealt in this work), but in the end of 2019 new nests were detected, highlighting the need for attention to the potential of *V. velutina* to rapidly colonise the British mainland, if control strategies are not timely applied (Keeling et al., 2017). As *V. velutina* gynes can fly over long distances (Monceau et al., 2015; Robinet, Suppo & Darrouzet, 2017) and as jump-dispersal mediated by humans also occurs (Robinet, Darrouzet & Suppo, 2019; Verdasca et al., 2021c), the non-invaded high-risk regions of south of Italy, Corsica, Croatia and Montenegro, should be vigilant. This is relevant, because in a previous work, we evidenced that in regions where the climatic conditions are suitable for *V. velutina*, motorways play an important role in accelerating the natural invasion dynamics of this invasive (Verdasca et al., 2021c). Independently of the colonization mechanisms, countries more at risk of being invaded should elaborate action plans focusing on biosecurity policies (particularly targeted to the interception of wooden products’ transportation and man-made goods associated with garden trade, due to the potential of these commodities to shelter hibernating queens) and addressing the expected impacts on native biodiversity, crop pollination, beekeeping activities and public health.

## Conclusions

Our approach of combining reciprocal distribution models with analyses of niche dynamics strengthens our predictions on the potential of *V. velutina* to continue its invasion process. With RDM we identified which areas are most sensitive to invasion and with niche analysis we confirmed (i) its ability to colonize different environmental envelopes from its native realized niche (evidence of a putative niche shift, especially in NW of Iberian Peninsula) and (ii) that the species is using the most common available conditions in Europe (hence less prone to stochastic events that can limit its expansion process). This approach is useful to forecast the future of the *V. velutina* expansion in western and central Europe and might be used with success for many other invasives.

## Supplemental Information

10.7717/peerj.13269/supp-1 Supplemental Information 1 Supplemental Tables and Figures

10.7717/peerj.13269/supp-2 Supplemental Information 2 *Vespa velutina* occurrences - 5 km resolution

## Additional Information and Declarations

### Data Availability

The following information was supplied regarding data availability: The vespa velutina data is available in the Supplementary File.
