# Supplementary material for: Contrasting patterns from two invasion fronts suggest a niche shift of an invasive predator of native bees
Source: PeerJ. 2022 May 10;10:e13269. doi: 10.7717/peerj.13269 (PMC9104094; doi:10.7717/peerj.13269)
Supplement: Supplemental Information 1 [file peerj-10-13269-s001.pdf]

PeerJ

Electronic Supplementary Material

Contrasting patterns from two invasion fronts suggest a niche shift of an invasive predator of native bees

Maria João Verdasca, Luísa Gigante Carvalheiro, Jesús Aguirre-Gutiérrez, José Pedro Granadeiro, Quentin Rome, Sébastien J. Puechmaille, Rui Rebelo, Hugo Rebelo

Corresponding author: Maria João Verdasca | E-mail : mjoao.v@gmail.com

Sources of Information

- ICNF - Instituto da Conservação da Natureza e das Florestas (Portuguese records);
- Bombeiros Voluntários de Viana do Castelo (Portuguese records);
- Ministerio de Agricultura y Pesca, Alimentación y Medio Ambiente (Spanish records provided on a 1 km x 1 km grid - we used the latitude and longitude of the centroid of each presence);
- INPN - Muséum National d'Histoire Naturelle in Paris (native and invasive French records);
- GBIF – Global Biodiversity Information Facility (native and invasive records with a geographical uncertainty below the resolution of our pixel size - 5km). Available at: www.gbif.org
- Life STOP vespa (italian records): Data available on the online website of the project at: https://www.vespavelutina.eu/it-it/ (Accessed: 1 September 2018).
- Data available in *Vespa velutina* in Europe - Google Maps 2017. Available at: https://www.google.com/maps/d/u/0/viewer?mid=1jRfoi4oF6GmiGRgbXuD71Qpbw8s&hl=en_US&ll=47.89184612561176%2C10.028028255145273&z=6 (Accessed: 1 September 2018).

Methodological details of modelling techniques

The GBM models were constructed by fitting 2500 trees and 3 cross-validations to identify the number of trees that produced most accurate predictions. For RF models 500 trees were used as the building criterion with a node size of 5. MaxEnt was run with linear, quadratic, product, threshold and hinge features.

To evaluate the importance of variables for determining the suitable environment biomod2 applies a randomisation procedure randomly shuffling the values of the focus environmental variable and fitting a model. Then the model predictions of the 'random' and original model are compared, and their correlation value is obtained. Then the variable importance equals 1 – correlation, so that unimportant variables have values as low as 0 (not important) and important variables have high values of up to 1 (highly important). We extracted the resulting variable importance values for all models created from biomod2.

Tables

Table S1 – Environmental variables with potential to affect the ecophysiology of *Vespa velutina*. The source where each variable was obtained, and its original resolution is indicated in the table.

Type	Variables	Original resolution	Source
Climatic	BIO1 = Annual Mean Temperature (ºC)	2.5m (~5km)	worldclim v.2
	BIO2 = Mean Diurnal Range (Mean of monthly (max temp - min temp) (ºC)	2.5m (~5km)	worldclim v.2
	BIO4 = Temperature Seasonality (standard deviation *100) (ºC)	2.5m (~5km)	worldclim v.2
	BIO5 = Max Temperature of Warmest Month (ºC)	2.5m (~5km)	worldclim v.2
	BIO6 = Min Temperature of Coldest Month (ºC)	2.5m (~5km)	worldclim v.2
	BIO7 = Temperature Annual Range (BIO5-BIO6) (ºC)	2.5m (~5km)	worldclim v.2
	BIO10 = Mean Temperature of Warmest Quarter (ºC)	2.5m (~5km)	worldclim v.2
	BIO11 = Mean Temperature of Coldest Quarter (ºC)	2.5m (~5km)	worldclim v.2
	BIO12 = Annual Precipitation (mm)	2.5m (~5km)	worldclim v.2
	BIO13 = Precipitation of Wettest Month (mm)	2.5m (~5km)	worldclim v.2
	BIO14 = Precipitation of Driest Month (mm)	2.5m (~5km)	worldclim v.2
	BIO15 = Precipitation Seasonality (Coefficient of Variation) (mm)	2.5m (~5km)	worldclim v.2
	BIO16 = Precipitation of Wettest Quarter (mm)	2.5m (~5km)	worldclim v.2
	BIO17 = Precipitation of Driest Quarter (mm)	2.5m (~5km)	worldclim v.2
	BIO19 = Precipitation of Coldest Quarter (mm)	2.5m (~5km)	worldclim v.2
Topography	DEM - Altimetry (m)	30s (1km)	Hydrosheds DEM
	Slope	30s (1km)	Hydrosheds DEM
	Northeness	30s (1km)	Hydrosheds DEM
	Eastness	30s (1km)	Hydrosheds DEM
Land cover	Distance to urban areas (m)	300m	ESACCI-LC_LCCS v2.0.7
	Distance to forest (m)	300m	ESACCI-LC_LCCS v2.0.7
	Distance to water (m)	300m	ESACCI-LC_LCCS v2.0.7

Note: Climatic data were obtained from WORLDCLIM at 2.5-arcmin resolution (Hijmans *et al.*, 2005), HydroSHEDS topographic data (Lehner, Verdin and Jarvis, 2006) were obtained at 30 s resolution and land-cover data were extracted from European Space Agency (2017) at a resolution of 300 m.

References

European Space Agency (2017) Land Cover CCI Product User Guide Version 2, CCI-LC-PUGV2. Available at: http://maps.elie.ucl.ac.be/CCI/viewer/download/ESACCI-LC-Ph2-PUGv2_2.0.pdf.

Hijmans RJ, Cameron S, Parra J, Jones P, Jarvis A (2005) Very high resolution interpolated climate surfaces for global land areas. International Journal of Climatology 25(15), pp. 1965–1978. doi: 10.1002/joc.1276.

Lehner B, Verdin K, Jarvis A (2006) HydroSHEDS Technical Documentation. World Wildlife Fund US, Washington, DC. Available at: http://hydrosheds.cr.usgs.gov.

Table S2 - Correlation matrix of the environmental variables that have the potential to affect the ecophysiology of *Vespa velutina* in Europe. Correlated variables (r≥0.70) are shown in grey.

Variables	bio1	bio2	bio4	bio5	bio6	bio7	bio10	bio11	bio12	bio13	bio14	bio15	bio16	bio17	bio19	dem	dfor	durb	dwat	northn	eastn	slope
**bio1**	1.00																					
**bio2**	-0.04	1.00																				
**bio4**	-0.33	0.66	1.00																			
**bio5**	0.54	0.69	0.54	1.00																		
**bio6**	0.77	-0.58	-0.81	-0.07	1.00																	
**bio7**	-0.19	0.86	0.93	0.70	-0.76	1.00																
**bio10**	0.82	0.34	0.26	0.89	0.31	0.36	1.00															
**bio11**	0.88	-0.37	-0.73	0.13	0.96	-0.60	0.47	1.00														
**bio12**	0.31	-0.25	-0.68	-0.21	0.55	-0.53	-0.09	0.57	1.00													
**bio13**	0.44	-0.36	-0.73	-0.16	0.68	-0.59	0.03	0.69	0.95	1.00												
**bio14**	-0.60	0.26	0.36	-0.23	-0.60	0.27	-0.42	-0.64	-0.22	-0.46	1.00											
**bio15**	0.57	-0.42	-0.70	-0.04	0.77	-0.57	0.19	0.78	0.69	0.86	-0.81	1.00										
**bio16**	0.43	-0.38	-0.77	-0.19	0.70	-0.62	0.00	0.70	0.95	0.99	-0.47	0.87	1.00									
**bio17**	-0.45	0.32	0.32	-0.11	-0.51	0.29	-0.27	-0.49	-0.05	-0.31	0.95	-0.71	-0.33	1.00								
**bio19**	0.42	-0.31	-0.76	-0.17	0.68	-0.60	-0.02	0.68	0.95	0.96	-0.44	0.83	0.98	-0.31	1.00							
**dem**	-0.44	0.04	0.10	-0.16	-0.34	0.14	-0.35	-0.32	0.20	0.15	0.01	0.07	0.13	0.07	0.08	1.00						
**dtrees**	0.01	0.03	0.16	0.07	-0.07	0.09	0.09	-0.09	-0.31	-0.29	0.06	-0.21	-0.28	0.00	-0.26	-0.25	1.00					
**durb**	-0.22	0.24	0.13	0.04	-0.26	0.21	-0.13	-0.20	0.11	0.04	0.16	-0.09	0.02	0.22	0.02	0.45	-0.18	1.00				
**dwat**	-0.11	0.07	0.11	0.04	-0.13	0.12	-0.04	-0.12	0.02	0.00	0.02	0.00	0.00	0.06	-0.01	0.23	-0.08	0.21	1.00			
**northn**	0.01	0.00	-0.03	-0.02	0.01	-0.02	-0.01	0.01	-0.01	-0.01	0.03	-0.02	-0.01	0.02	-0.01	-0.12	0.05	-0.05	-0.04	1.00		
**eastn**	-0.02	-0.01	0.05	0.02	-0.03	0.03	0.02	-0.03	-0.05	-0.04	-0.01	-0.02	-0.05	-0.01	-0.06	0.09	-0.03	0.03	0.01	0.02	1.00	
**slope**	-0.11	-0.12	-0.11	-0.12	-0.01	-0.07	-0.15	0.01	0.34	0.33	-0.17	0.27	0.31	-0.07	0.26	0.60	-0.27	0.27	0.12	-0.01	0.04	1.00

Table S3 - Correlation matrix of the environmental variables that have the potential to affect the ecophysiology of *Vespa velutina* in Asia. Correlated variables (r≥0.70) are shown in grey.

Variable	bio1	bio2	bio4	bio5	bio6	bio7	bio10	bio11	bio12	bio13	bio14	bio15	bio16	bio17	bio19	dem	dfor	durb	dwat	northn	eastn	slope
**bio1**	1.00																					
**bio2**	-0.16	1.00																				
**bio4**	-0.44	-0.05	1.00																			
**bio5**	0.67	-0.04	0.34	1.00																		
**bio6**	0.91	-0.25	-0.73	0.33	1.00																	
**bio7**	-0.42	0.22	0.95	0.38	-0.75	1.00																
**bio10**	0.76	-0.22	0.25	0.97	0.45	0.24	1.00															
**bio11**	0.91	-0.10	-0.77	0.32	0.98	-0.74	0.42	1.00														
**bio12**	0.20	-0.43	-0.64	-0.38	0.49	-0.75	-0.25	0.44	1.00													
**bio13**	0.26	-0.24	-0.68	-0.34	0.51	-0.74	-0.22	0.49	0.87	1.00												
**bio14**	0.01	-0.56	-0.04	-0.08	0.14	-0.19	0.00	0.05	0.54	0.14	1.00											
**bio15**	0.29	0.28	-0.47	-0.09	0.34	-0.39	-0.05	0.41	0.08	0.50	-0.66	1.00										
**bio16**	0.26	-0.25	-0.70	-0.36	0.52	-0.76	-0.23	0.50	0.90	0.99	0.17	0.48	1.00									
**bio17**	0.01	-0.54	-0.06	-0.08	0.14	-0.20	-0.01	0.05	0.56	0.15	0.99	-0.67	0.19	1.00								
**bio19**	-0.13	-0.42	0.07	-0.11	-0.02	-0.06	-0.08	-0.11	0.45	0.06	0.89	-0.72	0.10	0.91	1.00							
**dem**	-0.71	0.40	-0.18	-0.84	-0.47	-0.12	-0.90	-0.42	0.02	0.03	-0.14	0.08	0.04	-0.13	-0.01	1.00						
**dtrees**	0.10	-0.16	0.31	0.34	-0.03	0.27	0.35	-0.06	-0.26	-0.24	-0.06	-0.07	-0.25	-0.09	-0.09	-0.37	1.00					
**durban**	-0.11	0.16	-0.46	-0.47	0.10	-0.42	-0.46	0.13	0.32	0.34	-0.04	0.22	0.35	-0.01	0.00	0.45	-0.36	1.00				
**dwat**	-0.20	0.27	-0.23	-0.35	-0.08	-0.17	-0.40	-0.04	0.11	0.22	-0.16	0.18	0.21	-0.15	-0.11	0.44	-0.19	0.46	1.00			
**northn**	0.09	0.22	-0.07	0.07	0.07	-0.01	0.04	0.09	0.01	0.09	-0.09	0.13	0.07	-0.07	-0.06	-0.09	-0.02	0.06	0.03	1.00		
**eastn**	0.03	-0.04	0.11	0.11	-0.03	0.10	0.12	-0.03	-0.18	-0.18	-0.03	-0.10	-0.20	-0.05	-0.10	-0.02	0.06	0.05	-0.20	0.00	1.00	
**slope**	-0.28	0.15	-0.30	-0.54	-0.09	-0.29	-0.54	-0.07	0.21	0.32	-0.06	0.25	0.31	-0.05	-0.06	0.47	-0.40	0.34	0.24	0.02	-0.11	1.00

Table S4 – Ranges of the selected climatic variables used for modelling of the ecological niche of *Vespa velutina* in native and invasive distribution areas. The range of climatic variables used for each invasive population P1 (population from France and contiguous records) and P2 (population from NW of Iberian Peninsula) is also shown.

**Variables**	**Non-native Available Climate**		**Native Available Climate**		**Invaded niche**				**Native niche**	
	**Min**	**Max**	**Min**	**Max**	**Min P1**	**Max P1**	**Min P2**	**Max P2**	**Min**	**Max**
Bio1= Annual Mean Temperature (ºC)	-8.7	18.6	-13.5	28.9	3.5	15.9	10.1	16.7	4.4	25.3
Bio7 = Temperature Annual Range (BIO5-BIO6) (ºC)	12.3	33.5	9.1	41.9	15.7	30.0	14.9	26.2	14.6	35.6
Bio10 = Mean Temperature of Warmest Quarter (ºC)	-2.5	26.8	-7.0	32.4	10.8	22.9	15.7	24.1	13.0	29.4
Bio11 = Mean Temperature of Coldest Quarter (ºC)	-14.3	13.3	-22.7	27.5	-2.8	9.6	3.6	10.7	-8.8	21.7
Bio17 = Precipitation of Driest Quarter (mm)	10	562	0	773	6.5	294	46	161	16	493
Bio19 = Precipitation of Coldest Quarter (mm)	63	713	0	3228	104	425	246	644	21	495

Figures

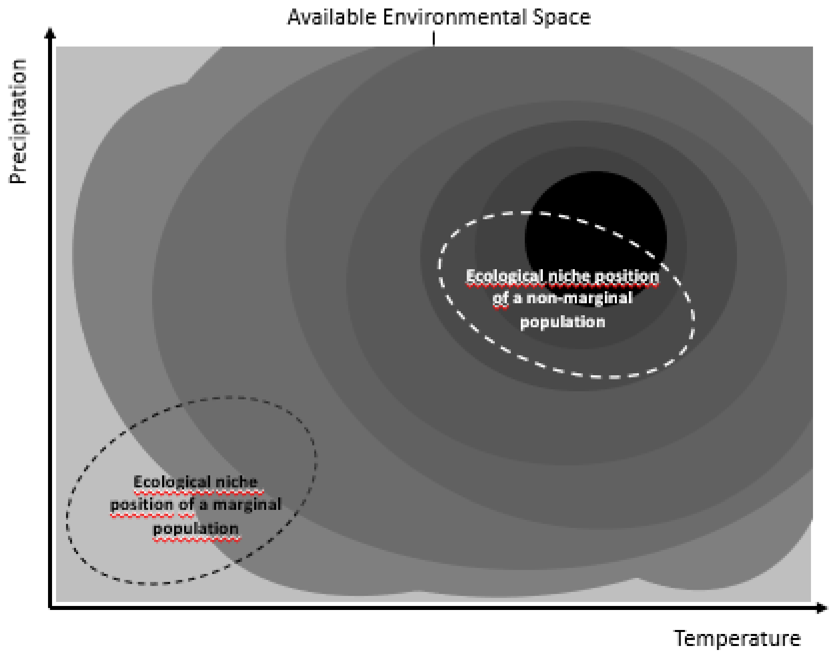

Fig. S1 - Schematic representation of the species' marginality concept. The large square represents the total available environmental space. The growing intensity of the colour is proportional to the frequency of the combined environmental conditions in a geographical area, from the less frequent or marginal environmental conditions (light grey) to the most common conditions (black). The dashed lines, black and white, represents the ecological niche position of a marginal and non-marginal population, respectively.

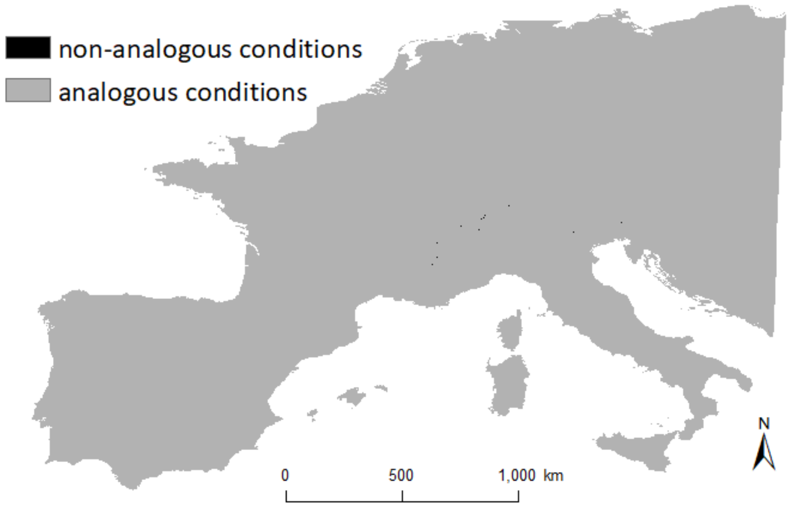

Fig. S2 - Comparison of the environmental similarity of variables to the environmental data used for training the model. Multivariate Environmental Similarity Surfaces (MESS) shows areas in grey, representing the regions with environmental similarity to the native range and areas in black (very small: 0.008% of the Europe area) having one or more environmental variables outside the range present in the training data, so predictions in those areas should be treated with strong caution.

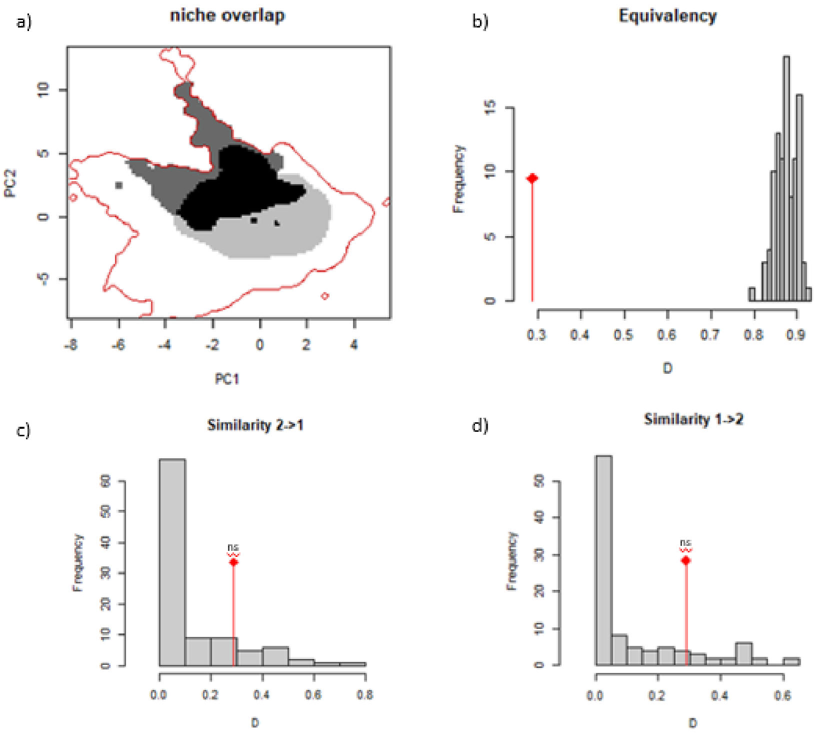

Figure S3 – Similarity and equivalence test between native and invasive distribution of P1. Visual representation of niche overlap between native and invasive ranges where the solid contour line illustrates the kernel density estimates corresponding to 100% of the available (background) environment (a); Histograms (b-c-d) show the observed niche overlap D between the two ranges (bars with a diamond) and simulated niche overlaps (grey bars) on which tests of niche equivalency (b), niche similarity of Europe-P1 to Asia (c), and niche similarity of Asia to Europe-P1 (d) are calculated from 100 iterations. The significance of the tests is shown (ns, non-significant).

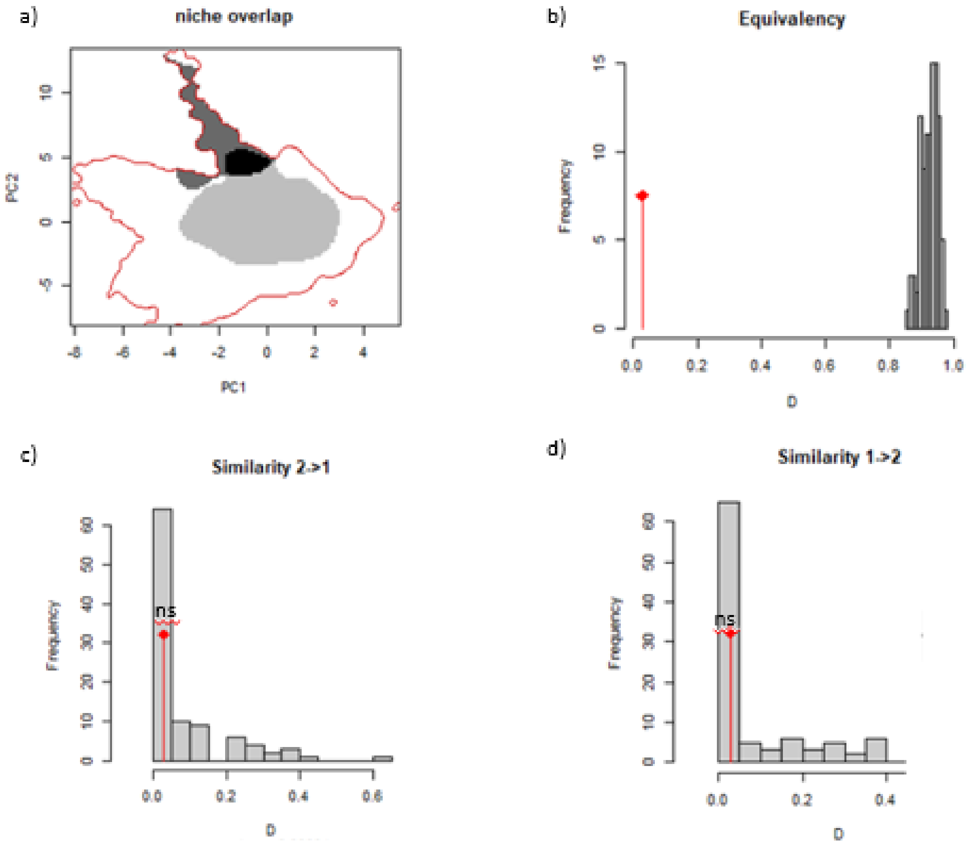

Figure S4 – Similarity and equivalence test between native and invasive distribution of P2. Visual representation of niche overlap between native and invasive ranges where the solid contour line illustrates the kernel density estimates corresponding to 100% of the available (background) environment (a); Histograms (b-c-d) show the observed niche overlap D between the two ranges (bars with a diamond) and simulated niche overlaps (grey bars) on which tests of niche equivalency (b), niche similarity of Europe-P2 to Asia (c), and niche similarity of Asia to Europe-P2 (d) are calculated from 100 iterations. The significance of the tests is shown (ns, non-significant).

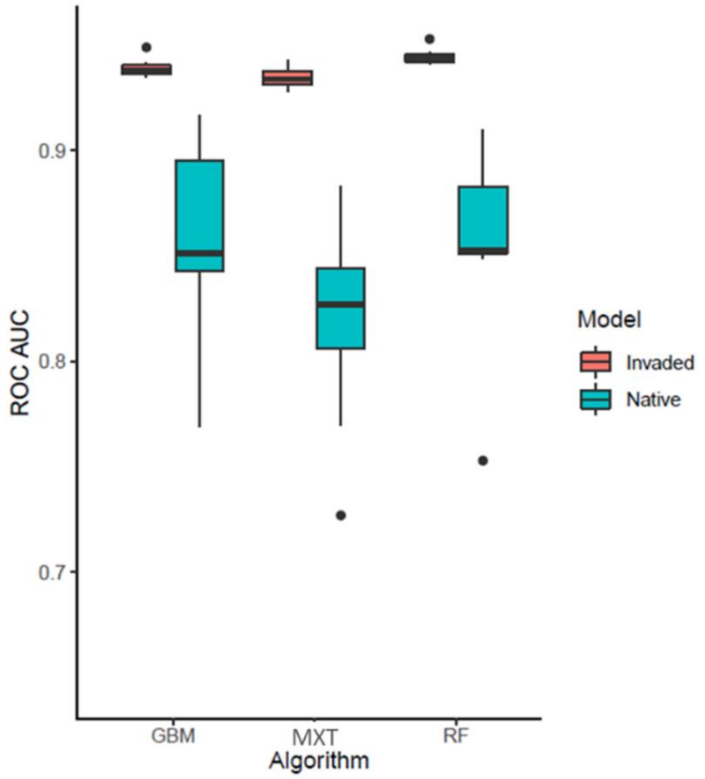

Fig. S5 – Variation of the Area Under the Curve (AUC) across the different modelling techniques.

a) b)

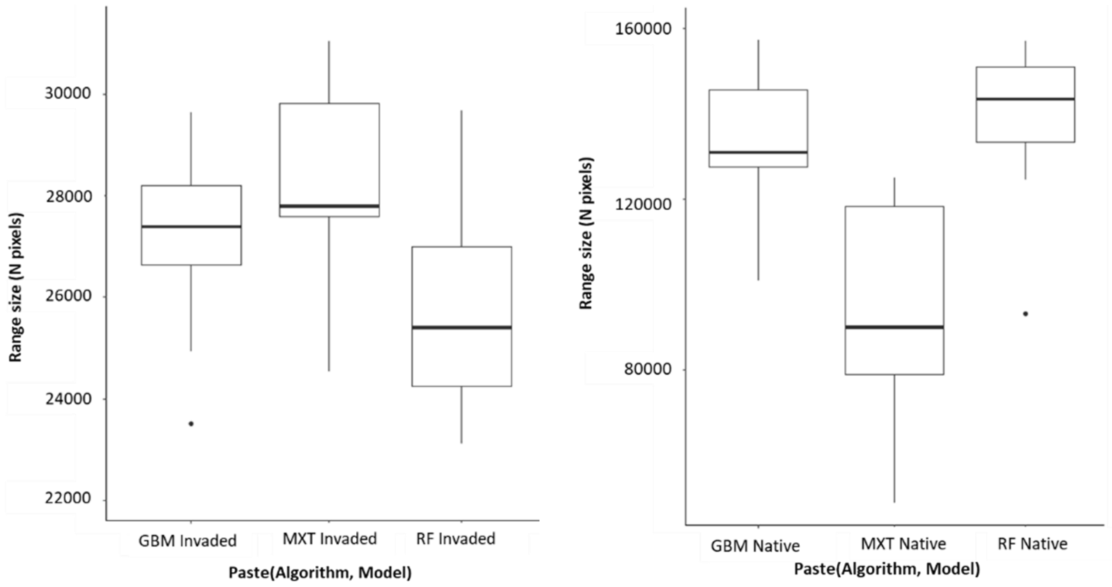

Fig. S6 – Variation of the predicted European range size with the different modelling techniques using the invasive (a) and native (b) models.

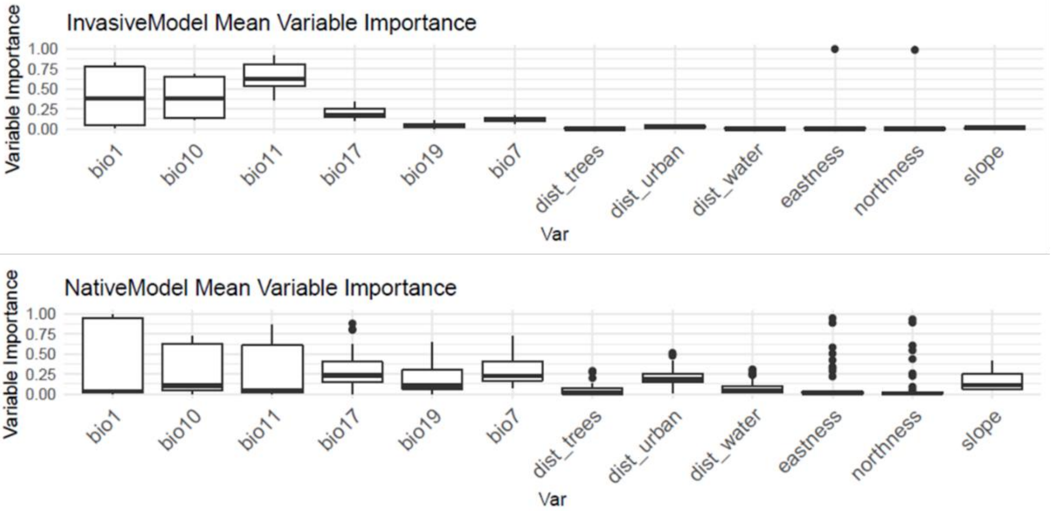

Fig. S7 – Variation of variable importance according to invasive and native models.
